# Common Elements Approaches to Implementation Research and Practice: Methods and Integration with Intervention Science

**DOI:** 10.1007/s43477-023-00077-4

**Published:** 2023-03-22

**Authors:** Thomas Engell, Nicole A. Stadnick, Gregory A. Aarons, Miya L. Barnett

**Affiliations:** 1 Centre for Child and Adolescent Mental Health, Eastern and Southern Norway, Gullhaugveien 1-3, 0484 Oslo, Norway; 2 Department of Psychiatry, University of California San Diego, La Jolla, CA 92093 USA; 3 Child and Adolescent Services Research Center, San Diego, CA 92123 USA; 4 University of California San Diego Altman Clinical and Translational Research Institute Dissemination and Implementation Science Center, La Jolla, CA 92093 USA; 5 Department of Counseling, Clinical, & School Psychology, University of California, Santa Barbara, CA 93106-9490 USA

**Keywords:** Common elements, Implementation elements, Evidence synthesis, Pragmatic implementation science, Precision tailoring

## Abstract

We propose that *common elements approaches* can advance implementation research and practice and facilitate pragmatic use of intervention and implementation evidence. Common elements are practices or processes frequently shared by interventions or implementations. Traditional common elements methodologies use synthesis, distillation, and statistics to describe and evaluate the merit of common ingredients in effective interventions. Recent developments include identifying and testing common configurations of elements, processes, and context variables across the literature of effective interventions and implementations. While common elements thinking has grown popular in intervention science, it has rarely been utilized in implementation science, and specifically, combined with the intervention literature. The goals of this conceptual methodology paper are to (1) provide an overview of the common elements concept and how it may advance implementation research and usability for practice, (2) give a step-by-step guide to systematic common elements reviews that synthesizes and distills the intervention and implementation literature together, and (3) offer recommendations for advancing element-level evidence in implementation science. A narrative review of the common elements literature was conducted with attention to applications to implementation research. A six-step guide to using an advanced common elements methodology was provided. Examples of potential results are presented, along with a review of the implications for implementation research and practice. Finally, we reviewed methodological limitations in current common elements approaches, and identified steps towards realizing their potential. Common elements methodologies can (a) synthesize and distill the implementation science literature into practical applications, (b) generate evidence-informed hypotheses about key elements and determinants in implementation and intervention processes and mechanisms, and (c) promote evidence-informed precision tailoring of intervention and implementation to context. To realize this potential, common elements approaches need improved reporting of details from both successful and unsuccessful intervention and implementation research, more data availability, and more testing and investigation of causal processes and mechanisms of change from diverse theories.

**Supplementary Information:**

The online version contains supplementary material available at 10.1007/s43477-023-00077-4.

Implementation processes encompass a range of unique and shared strategies and tools that are used or facilitated by people or systems to support implementation of an innovation (Proctor et al., 2013). Whether they are events, ongoing activities, interactions, or phenomena, implementation processes can be disentangled into *elements* of implementation that are present, occur, or emerge in time and space. These elements may or may not influence implementation mechanisms and outcomes in linear and non-linear ways. What we term *elements* of implementation can include what implementation science theorizes as discrete implementation strategies (Powell et al., 2015), implementation determinants (Nilsen & Bernhardsson, 2019), implementation competencies (Metz et al., 2021), or any other relevant *part* of implementation processes. Implementation science has largely adopted conceptualizations of discrete implementation strategies as the actionable activities and events we use to facilitate implementation (Powell et al., 2015), similar to what intervention science conceptualizes as discrete practice elements in interventions (i.e., “we define a practice element as a discrete clinical technique or strategy used as part of a larger intervention plan”; Chorpita et al., 2005). Subsequently, we tend to explain implementation in terms of regulatory causal pathways where implementation strategies assert effects on determinants and outcomes through causing mechanisms of change (Lewis et al., 2018).

Some implementation strategies are relatively “static” and granular, such as a calendar reminder or using an implementation checklist. However, other strategies may be more dynamic, emerging, and ecological processes that work as several interconnected elements when they facilitate implementation, such as tailoring to contextual needs, co-creation, leadership and organizational development to improve implementation climate, or learning collaboratives. The interconnected elements that make up these dynamic strategies are not necessarily just disentangled parts of what we typically describe as a discrete implementation strategy. These elements may, for instance, include specific contextual circumstances or properties, relationships, cultural values, and competencies in actors in implementation—and they may be the elements of the strategy that catalyze or determine causal effects, and not necessarily the discrete events or activities (Wagner, 1999). Thus, when implementation processes drive change, the causes can also be attributed to configurations and patterns of different types of elements of implementation acting together, or on each other, as dynamic processes or systems (Braithwaite et al., 2018; Sterman, 2002). Different conceptualizations and causal theories do not necessarily exclude each other. Instead, different explanations of observations may complement each other and enrich our view of how implementation works. However, doing so necessitates considering the relevant elements that can be at play. With the vast amount of potentially relevant elements in implementation, we may benefit from scientific tools that help us narrow them down to those most likely to be influential in a given context. Common elements approaches offer methods for such distillation.

Several taxonomies, reporting standards, and frameworks exist across the implementation literature to help specify elements and outcomes (e.g., the ERIC taxonomy, Powell et al., 2015; the AACTT-framework, Presseau et al., 2019; the Behavior change technique taxonomy, Michie et al., 2013; the “name it—define it—specify it”—recommendations, Proctor et al., 2013; StaRi, Pinnock et al., 2017; TIDieR, Hoffmann et al., 2014; and AGReMA, Cashin et al., 2022). Implementation frameworks and syntheses based on comprehensive theoretical and empirical work arguably articulate elements that commonly are important for implementation (e.g., Cane et al., 2012; Damschroder et al., 2009; Moullin et al., 2019; Fixsen et al., 2005; Greenhalgh et al., 2004). However, the importance or utility of one or more common elements can depend on other elements, such as contextual and process factors. These factors suggest the need to ask more specific questions: When and under what circumstances do certain implementation strategies tend to work, and when do they not? Do they work as discrete strategies, or do they need to be interconnected parts of a blended, ecological, or sequential strategy? When do specific contextual determinants implicate a particular combination of strategies, and does it depend on the characteristics of the innovation being implemented or the competencies of implementation practitioners? What are the core elements of effective implementation strategies, and what strategies tend to be adaptable and when? Answers to such questions are complex and challenging to model generically, in part because they have conditions attached to them and constantly change. However, more advanced common elements methodologies can potentially provide useful answers, and in this paper, we propose language, methodology, and recommendations for the field to realize such potentials.

Common elements approaches have established a foothold in research on psychosocial, academic, and physical interventions (i.e., intervention science), and the concept of common elements proliferated as an approach to identify intervention practices common to effective interventions (Chorpita et al., 2005). This conceptual methodology paper discusses how the concept also applies to implementation science and how developments in common elements conceptualization and methods can facilitate practical implications for both intervention and implementation research and practice. We provide a step-by-step guide for using an advanced common elements methodology to synthesize and distill the intervention and implementation science literature into evidence-informed hypotheses about what, how, when, and where interventions and implementations tend to succeed. Our specific goals are to:

1. Provide a narrative review of the common elements concept and how its applications may advance implementation research and usability for implementation practice.
2. Provide a step-by-step guide to a systematic common elements methodology that synthesizes and distills the intervention and implementation literature together.
3. Offer recommendations for advancing element-level evidence in implementation science.

## A Narrative Review of the Common Elements Concept

### Similarities Between Intervention Science and Implementation Science

*Intervention science* is an umbrella term for research focused on human interventions within health and social sciences. Complex health and social interventions (Hawe et al., 2004) across service systems are likely to share commonalities such as practices, processes, structures, and delivery formats. Complex interventions (e.g., evidence-based programs) often also promote the same competencies in practitioners. There are debates about whether the focal points of intervention research should be on intervention techniques (e.g., discrete practice elements; McLeod et al., 2017), common factors (e.g., therapeutic alliance, empathy, and expectations; Wampold, 2015), therapist skills (e.g., interpersonal capacities, cognitive processing, self-assessment; Heinonen & Nissen-Lie, 2020; Hill et al., 2017), principles- and processes of change (e.g., process-based therapy; Hofmann & Hayes, 2019), or more complex manualized programs (e.g., Parent Child Interaction Therapy; Eyberg & Funderburk, 2011), even though scholars generally tend to recognize that all of the above more or less are relevant for the outcomes of interventions (Mulder et al., 2017).

Implementation science replicates intervention science in many regards, partly because they share philosophical and theoretical foundations (Engell, 2021). For example, blended implementation strategies resemble complex health programs; discrete implementation strategies resemble practice elements and techniques; implementation competencies resemble common factors and therapist skills; implementation drivers and processes resemble intervention principles and processes of change. There are also similar debates about what is more critical for successful implementation outcomes (e.g., a greater focus on discrete implementation strategies, blended strategies, dynamic systems, implementation competencies and relational skills, or guiding frameworks). Intervention and implementation science also share similar and connected research-to-practice gaps and call for the same type of research and methods to close them (e.g., more pragmatic applications of scientific evidence, more contextual alignment, and advancing training; Lyon et al., 2020b; Westerlund et al., 2019). These similarities indicate that methodological innovations in intervention science, such as common elements approaches, are likely to generalize to the younger but rapidly developing implementation science. However, instead of implementation research having to replicate the limitations that intervention research has encountered, such as limited implementability in interventions (Lyon et al., 2020a) and lacking understanding of how intervention practices work (e.g., active ingredients and mechanisms of change; Huibers et al., 2021; Kazdin, 2009), early accommodation of advanced common elements approaches may help address and avoid these limitations. There may also be benefits to purposefully connecting intervention and implementation science together in conceptualizations of common elements.

### Common Elements Approaches in Intervention Science

The many shared features between complex mental health interventions led some scholars to argue that the elements found common across several effective interventions are more likely to contribute to positive outcomes than less common elements (Chorpita et al., 2005). That is, they are more likely to be the *active* or *potent* ingredients of interventions contributing to positive outcomes, or what Embry and Biglan referred to as evidence-based kernels (Embry & Biglan, 2008). Simply defined, common elements are practices or processes frequently shared by a selection of interventions (Engell et al., 2020). Depending on the philosophical orientation and the methodology used to identify them, common elements are assumed to have certain qualities or characteristics (e.g., active ingredients, essential elements, evidence-informed elements). Common elements approaches disentangle complex interventions into discrete elements and then describe or evaluate the relative merits of common elements across the scientific literature using varying levels of refinement.

Typical common elements approaches include mapping and distilling practice elements through literature reviews (Garland et al., 2008), identifying (potentially) active ingredients (e.g., Abry et al., 2015), using common elements for dissemination and implementation (e.g., Centre for Evidence & Implementation, 2020), and using identified common elements to inform the design or re-design of interventions (e.g., Engell et al., 2021). Originally, the analytical approaches used in common elements work were considered purely descriptive (Chorpita et al., 2007). However, work on common elements and similar concepts has developed in several directions. Traditional common elements methods are usually either based on expert opinions (e.g., Delphi methods; Garland et al., 2008), descriptive commonalities or frequencies (e.g., in systematic reviews; van der Pol et al., 2019; or practice-based observation; Hogue et al., 2019), statistical testing of associations (e.g., meta-regressions; Leijten et al., 2019), or combinations of these methods. See Leijten et al. (2021) for a scoping review of methods to evaluate active ingredients in interventions. Recent developments involve methods and statistics for reviewing and testing conjunctions of client characteristics, practice elements, and delivery formats (Furukawa et al., 2021), and reviewing combinations of different types of intervention practices, processes, contextual characteristics, and discrete implementation strategies to identify configurations that tend to work (Engell, 2021). These different but related methodologies share common goals of generating hypotheses about the more likely effective intervention contents, and subsequently fine-grained testing of intervention contents and mechanisms (Chorpita et al., 2011; Engell et al., 2020).

Common elements approaches have, in part, evolved as a response to frequently encountered issues with the implementation of evidence-based psychosocial care in the form of comprehensive programs with standardized protocols and models of implementation. The issues raised include inflexible programs and standardized protocols lacking contextual fit and sensitivity to unpredictable variation and change (Stirman & Comer, 2018). Comprehensive programs can also be demanding to implement, sustain, and coordinate in the multiple numbers needed to cover the totality of client needs that organizations providing psychosocial care encounter (Chorpita & Daleiden, 2018). Common elements approaches have been linked with a range of benefits for implementation that can counter such issues (Barth et al., 2020; Bolton et al., 2014; Conroy et al., 2019; Murray et al., 2020; Weiss et al., 2015; Weisz et al., 2012). For instance, increasing our understanding of effective intervention elements, and the mechanisms they potentiate, can focus implementation of interventions on the elements most likely to contribute to positive outcomes and discarding elements that are likely unnecessary or harmful. Doing so may reduce the complexity of interventions and increase their implementability as well as efficiency and effectiveness. Combining element approaches with research using multiple causal theories can also help increase our understanding of the *how*, *why*, and *when* interventions tend to work, to complement the primacy to the *whether* and *how often* efficacy/effectiveness questions that have traditionally dominated the evidence-based paradigm (Engell, 2021). Such understandings may help reconcile static population-based evidence with implementation of more personalized, ecological, and dynamic approaches to care (Engell et al., 2021), and unveil adaptations to interventions that tend to be favorable under different circumstances (e.g., Park et al., 2022). Common elements can also be “building blocks” to be reassembled and tailored into new or adapted interventions or other models and forms of implementation in practice (Chorpita et al., 2021; Engell et al., 2021). The common elements concept yet has important limitations that we will discuss, but it appears useful towards illuminating what tends to drive effective interventions and facilitating opportunities to make intervention evidence implementable and accessible to complex care settings.

### Common Elements Logic and Language

There is no agreed-upon nomenclature in common elements research, and many terms are used interchangeably and differently. In our common elements work, we review and synthesize intervention and implementation elements together, and to do so, we use a language founded in established logic about the relationships between “parts” and “wholes” to make the common elements concept itself as atheoretical as we can (Engell, 2021; Varzi, 1996). That is why generic terms such as elements, components, and processes are preferred over more semantic terms such as discrete strategies or competencies when describing the concept and planning reviews. This enables us to systematically review and synthesize research from a wide range of theoretical perspectives, as well as reduce the risk of actively or unknowingly excluding relevant elements of the intervention or implementation. We can, of course, apply more theory in our choices of taxonomies when conducting the reviews or explaining their results. Table 1 provides a glossary of terms for common elements research and our understanding of what makes elements “core,” “common,” “evidence-informed,” and “evidence-based.” We explain in terms of implementation elements, but the logic is the same for intervention elements.

**Table 1:** Explanations of key terms used in common elements thinking and research

Term	Explanation
(Implementation) Elements	An implementation element is a generic term for any meaningfully distinguishable *part* of implementation at any level of *discreteness*. An element is a part of a whole that in itself also is a meaningful whole* *Example:* A provider’s self-efficacy towards an intervention may be an element of an implementation mechanism
(Implementation) Components	An implementation component is any part or ingredient of an implementation element. A component is not necessarily in itself a meaningful whole. We use the term when a second level of discreteness is needed, or to denote parts that depend on other parts to compose a whole *Example:* A brainstorm session for implementation barriers as part of a readiness assessment
Types of elements and components: Practice (x) Process (y) Context (z)	We categorize types of elements and components into: Practice elements/components (x) = things people do Process elements/components (y) = how people do things or how things unfold or emerge Context elements/components (z) = circumstances in which things are done, unfold or emerge *Example:* Conducting a readiness assessment (x) in facilitated collaboration (y) at a local community mental health clinic (z) using workshops (y) and a professional facilitator (z), with trust emerging among partners through value-based collaboration (y)
Core elements/components	Core elements are indispensable parts of a whole, for instance, a particular practice element in an implementation strategy. Without the practice element, the implementation strategy would be incomplete or something else. Preferably theoretically and empirically justified *Example:* A core element of the implementation strategy audit and feedback would be providing feedback
Common elements	Meaningful parts of wholes that are frequently shared by wholes. Common implementation elements can be practices or processes frequently shared among implementation strategies used in a selection of implementation studies *Example:* Role-play, modeling, and video review are common elements of consultation and supervision in cognitive-behavioral therapy interventions
Evidence-informed common elements	Meaningful parts frequently shared by empirically supported wholes (i.e., evidence-based interventions or evidence-based implementation strategies). Degree of ‘evidence-informedness’ may depend on for instance frequency, stability, and intensity in studies effectively improving a specific outcome accounted for frequency, stability, and intensity in ineffective or iatrogenic/harmful studies for the same outcome *Example:* The discrete implementation strategies quality monitoring and ongoing consultation are evidence-informed common elements for implementing reading interventions for children at risk for academic problems (Engell et al., 2020)
Evidence-based elements	Elements consistently demonstrating causal contribution to outcomes in experimental testing (causality can be inferred on the level of elements, not complex packages of them) *Example:* Audit and feedback is a multi-element implementation strategy that may be the closest example in the implementation literature, although effects tend to vary (Ivers et al., 2012; Tuti et al., 2017)

*What constitutes a *meaningful whole* will likely depend on context and perspectives on theory, and can also be informed by empirical support

A key initial step in several common elements approaches is deconstructing “the thing” to be implemented (e.g., the clinical intervention, program, or policy implemented) or “the stuff that we do to get people and places to do the thing” (implementation strategies; Curran, 2020) into its smaller meaningful entities (e.g., elements and components). Deconstruction is done to discern all the *parts/ingredients* that go into the “thing” so that we can use various methods to gain understanding about their contributions to outcomes. These contributions can be as discrete parts and sets of parts (e.g., when the thing is a sum of one or more active parts/ingredients). They can also be as parts of more ecological contribution (e.g., when the thing or system is more than the sum of its parts), or as parts in other meronymic relations (i.e., the relationships between parts and wholes; Varzi, 1996), such as for instance how elements may have dispositional causal powers that may be “triggered” by specific configurations of elements (i.e., “dispositional partner elements;” Anjum & Mumford, 2018). The appropriate level of deconstruction can depend on the objectives of the work and perspectives/theory on the nature and composition of the “thing.”

### Common Elements Approaches Applied to Implementation Science

Implementation science is in the early stages of establishing an evidence-base for implementation comparable to that in intervention science. As noted in Table 1, in terms of evidence for implementation strategies, audit and feedback is an empirically supported and relatively more “unpacked” implementation strategy to date (Brown et al., 2019; Ivers et al., 2012; Tuti et al., 2017). However, the common elements concept can be applied to other implementation strategies, guiding implementation frameworks, implementation competencies, training curriculums, and the literature on barriers and facilitators—both separately and conjunctively. Note that even though we suggest that the active content of implementation processes (i.e., elements) can go beyond discrete implementation strategies, element-level evidence would likely identify practice elements within discrete implementation strategies that are commonly key elements of implementation processes that succeed. By enhancing the accommodation of such element-level evidence in implementation science now in its early beginnings, we may avoid some of the limitations common elements work faces in intervention science today that holds detailed evidence back, such as insufficient reporting of details about interventions and implementation processes.

Methods that resemble common elements approaches have already had pragmatic and valuable implications for implementation research and practice. For example, the Expert Recommendations for Implementing Change (ERIC) project (Powell et al., 2015) used Delphi methods and concept mapping to discern the discrete implementation strategies that a group of implementation science experts identified as most important and feasible (Waltz et al., 2015)*.* Common elements methodologies can build on such taxonomies, disentangle them further, review large accumulations of the implementation literature at a refined level of detail, and extract the elements, components, and configurations most commonly used in successful implementation in specific contexts and circumstances. For instance, using frequency counts, we can discern the most common elements and configurations and then use meta-regression analyses to test how they are associated with implementation outcomes and/or clinical outcomes. Some of these common elements may be discrete implementation strategies as per the ERIC taxonomy, some may be more granular, some may tend to be interconnected with specific determinants, and some may be important parts of implementation that implementation science has yet to theorize.

In our common elements work, we found current taxonomies in intervention and implementation science helpful for operationalization and categorization of elements, but frequently also insufficiently detailed for fine-grained deconstruction. Table 2 shows an example from a common elements review of academic interventions and their implementation strategies (Engell et al., 2020) where we chose a discrete parts approach and used the ERIC taxonomy to code conjunctions between intervention elements, implementation elements, and contextual characteristics. During coding, we found that several discrete implementation strategies varied considerably in activities and how these activities were practiced. For instance, the level of refinement for the implementation strategy “*make training dynamic”* was insufficient to discern the differences in dynamic training activities used by interventionists (e.g., dynamic due to use of role plays, use of feedback, combining several activities, or other interactive activities). To capture more of the variation, we further deconstructed the strategy into more discrete elements for coding. However, precise details were difficult to ascertain because of limited descriptions of the training programs in the studies. More detailed reporting in primary studies, for instance, if they adhered to standards of reporting implementation strategies (Presseau et al., 2019; Proctor et al., 2013) would likely provide a more accurate, detailed, and exhaustive list and we would also be able to code more of their processual aspects (e.g., time and intensity, timing, sequencing, actors).

**Table 2:** Deconstruction of the implementation strategy “make training dynamic”

Discrete implementation element	Definition
Training using role play	Actively engaging trainees in changing their behavior to act out a role for the purpose of learning
Interactive training	Learning occurs through mutual actions between trainers and trainees. In interactive training, teaching and learning are interrelated, and trainees learn through a process of reflection through personal experience
Training with feedback	Any form of training with real-time feedback on performance or behavior from a trainer or peer
Combination of multiple dynamic training techniques with no specified order	Any form of training where two or more defined training techniques are combined (e.g., didactic + role play) without specifically describing how they are combined
Other dynamic training techniques	Any form of dynamic training not applicable to the training strategies above

An example of how common elements thinking can be used to open the “black box” of implementation can be found in Albers et al.’s (2021) integrative review of common implementation strategies used by professionals providing implementation support (implementation support practitioners [ISPs]). By looking at a large number of implementation studies across sectors, settings, and study designs, they first identified the most commonly used discrete implementation strategies by ISPs to support implementation, coded per the ERIC taxonomy. Next, they identified the most common activities and techniques (i.e., elements and components) used to carry out these discrete implementation strategies. They observed that the elements within each discrete implementation strategy varied considerably which raises the question of whether some elements are likely to be more useful or effective than others. They also observed that ISPs nearly always combined several implementation strategies, which raises questions about which combinations and their sequencing are most likely to be useful. Common elements approaches can help answer such questions.

A recent review by Tugendrajch et al. (2021) demonstrates the feasibility of descriptive common elements approaches in implementation science by reviewing the evidence for clinical supervision. First, they discerned the common elements of three different professional guidelines for providing clinical supervision of trainees and identified 17 elements that were common across the three guidelines. Next, they reviewed the literature to identify how the inclusion of these common elements in supervision was associated with therapist and client outcomes in therapy. They found that certain supervision elements (e.g., modeling ethical practices and documentation of supervision) were used in supervision with nearly exclusively positive outcomes, while other elements (e.g., self-evaluation and goal setting) were used in supervision with mixed outcomes. They also identified a general lack of attention to providing multicultural supervision, even though it is recommended in professional guidelines. Their results were, however, limited by scarce reporting of supervision details and potential publication bias. Despite the limitations, Tugendrajch et al. (2021) exemplify how common elements approaches not only operationalize the content of implementation strategies, but pragmatically review what content that tends to be included when strategies work, associations with their use across the literature, and identify content-specific knowledge gaps. However, the potential of common elements approaches stretches beyond such mapping, albeit requires more experimental primary studies, and more use of current and new reporting standards and data availability to realize its potential.

## Methods for Distilling and Testing Common Elements in Interventions and Implementations

Instead of using guidelines as the source of defining common elements, we could use a common elements approach across the extant literature on interventions and implementations. Supplementary file 1 provides a detailed step-by-step guide to how such a common elements approach can be conducted when sufficient data are reported and available. As an example, we explain how the approach could be used to review common elements of external consultation as an implementation strategy for psychosocial interventions (i.e., facilitation from outside of an organization or clinical unit; Nadeem et al., 2013). This approach will also discern the specific contexts and circumstances in which these common elements are used successfully and unsuccessfully. In recent common elements reviews that include coding common implementation elements (Engell et al., 2020; Helland et al., 2022), we have found that insufficient implementation data reported from intervention and implementation studies limit the full potential of conducting a review at this level of detail. For instance, in a review of common combinations of practices, processes, and implementation elements in academic interventions, we identified 62 practice elements, 49 process elements, and only 36 implementation elements (Engell et al., 2020). However, with the growing detail and use of reporting standards and data sharing, the feasibility and utility of such reviews will rapidly grow. The results from such reviews can inform evidence-informed hypotheses about key elements and mechanisms in successful implementation of interventions, identify detailed evidence gaps, and provide specific practical implications for intervention and implementation researchers and practitioners.

The approach we present is based on a systematic common elements-review methodology developed by Engell et al. (Engell et al., 2020), which has been used in different reviews of interventions in recent years (Helland et al., 2022; Mellblom et al., 2023; Winje, 2019; Bækken, 2021). We acknowledge that other methodologies can also be used, and we direct interested readers to the work of Chorpita and Daleiden (2009), Leijten et al. (2019), and McLeod et al. (2017) for prominent examples. Also, although it is not explicitly a common elements methodology, we acknowledge the approach Brown et al. (2019) took to unpack core elements and mechanisms of audit and feedback by systematically reviewing and analyzing qualitative studies. Using theory-based meta-review methods, with similarities to common elements reviews, Brown et al. systematically coded and synthesized qualitative studies of audit and feedback into a comprehensive theory of how audit and feedback works and elements and factors that influence their effects. We chose the common elements review methodology because it is, to our knowledge, the only methodology that purposefully connects common intervention elements, implementation elements, and context elements. Table 3 is a short summary of key steps in this advanced common elements review methodology based on a manual available in Engell et al. (2020). Elaboration of steps and practical advice for use are available in supplementary file 1.

**Table 3:** Summary of step-by-step guide to advanced systematic common elements review methodology

Step	Description
1	Study selection from systematic reviews or databases • Define systematic review criteria for identifying studies to code in common elements matrixes or databases • Literature search and selection following standards of high-quality systematic reviews (Higgins & Green, 2011)
2	Gather material, information, and data from included studies • Scientific papers and supplementary files, intervention manuals, implementation plans, data openly shared from the study, dissertations, research reports, and gray literature
3	Prepare and pilot the coding system • Create a conceptual framework and system for coding. Specify and define all elements, components, determinants, and outcomes anticipated as relevant. The deconstruction explained on page 9–10 is central to this process and includes making conscious decisions about theory. Use existing ontologies and taxonomies when relevant, deviate and report when appropriate • Use an iterative data-driven process of piloting for construct validity and coding reliability, and be inclusive rather than exclusive about elements/codes at this stage • Prepare coding matrices, a database, or other systems that will organize and store the coded information appropriately for conducting planned algorithms and analyses. A key feature needed for the method presented here is a network architecture (i.e., the ability to track each code and each combination or branch of codes back to its origin study and its specific outcomes) while at the same time collate codes from several studies together • Prepare for the opportunity to add unanticipated codes during coding. Inductive coding can help identify potentially meaningful novelties that do not fit existing theories and taxonomies and reduce biases towards “popular” elements (Engell et al., 2020)
4	Coding iterations • All study materials are coded by at least two independent coders, and coding conflicts are resolved through discussions or with a supervisor • Coding metrics are recorded to test coding reliability and inform revisions to the coding system • Iterative coding may be necessary if unanticipated elements are identified during coding and included in the coding system
5	Apply algorithms to identify common elements and combinations • Frequency-based algorithms to identify common elements and common combinations of elements • Algorithms for specific configurations of common elements • Calculate adjusted frequency values of common elements and combinations for each outcome and configurations of interest (e.g., inclusion in effective interventions/implementations accounted for inclusion in ineffective and iatrogenic) • Formulate hypotheses based on common elements, combinations, and configurations
6	Statistical analyses • See Leijten et al. (2021) for review of typical analytical strategies • Three-level meta-regression analyses (Assink & Wibbelink, 2016) to test how the inclusion and exclusion of common elements and common configurations are associated with effect sizes for different outcomes and how other elements moderate associations. Freely available shiny app in R developed by Wentzel-Larsen available here (Wentzel-Larsen, 2021) • Component Network Meta-Analysis (cNMA) to estimate the relative effectiveness of elements and combinations across studies. Can be conducted using Bayesian statistics or frequentist methods (Seide et al., 2020). See Pompoli et al. (2018) for analytic strategy and example application • Coincidence analyses (CNA, Baumgartner & Ambühl, 2020), a configurational-comparative method that can test causal regularities based on set theory combinations (i.e., conjunction, disjunction, and negation) of coded elements and characteristics • The perhaps most promising application of statistical analyses in this step is likely collating mega-reviews or continuously feed coded data into big data ecosystems with network architecture designed for machine learning. The application of machine learning and other artificial intelligence (AI) methods is discussed later in the article

Some common elements reviews restrict the study selection to effective studies or interventions that have outperformed a comparison condition (i.e., “winning interventions,” Chorpita & Daleiden, 2009; Barth et al., 2014; Okamura et al., 2020). Although doing so can be appropriate depending on the aims the review (e.g., describe the literature on effective interventions), as a general principle we recommend not excluding ineffective or less effective experimental conditions because it excludes information relevant for interpretation. As demonstrated in a common elements review of academic interventions (Engell et al., 2020), highly common elements in effective interventions may also be common in ineffective or less effective interventions. Thus, excluding studies without positive effects or with iatrogenic effects may skew results (e.g., popularity bias, Engell et al., 2020). Further, as recently demonstrated by Solheim-Kvamme et al. (2022), highly common elements of winning interventions may not necessarily be more associated with intervention effects than less common elements when their inclusion in ineffective and less effective interventions are statistically accounted for.

## Results from Integrated Common Elements Reviews

Table 4 summarizes the type of results we could potentially extract from a common elements review of above 100 richly reported intervention and implementation studies where external consultation was experimentally tested or used. The most basic algorithms in Step 5, calculating frequency values of singular elements, provides information about the *if* and *how often* common elements are used in implementations and interventions producing effects, accounted for use in ineffective implementations/interventions. The more advanced algorithms, calculating frequency values for common combinations of practices, processes, and context provide more information about the *how* and *when* of successful implementation and intervention*.* For instance, we can extrapolate answers to specific questions such as: What are the most commonly successful consultation elements used in implementation of transdiagnostic mental health interventions for children ages 6–12 in community clinics, how are these elements most commonly used successfully, and which implementation determinants most commonly facilitate or inhibit successful implementation when these consultation elements are used? Combined, the results can be formulated as evidence-informed hypotheses and/or implications about key elements, mechanisms, or processes in interventions and implementations. Further, some of these hypotheses can be tested using the analyses in step 6, and strengthen or weaken their “evidence-informedness” and implications for experimental testing or use in practice.

**Table 4:** Potential results from the example review of consultation strategies

1	A descriptive overview of the most commonly used consultation elements in implementation processes with successful outcomes, adjusted for use in implementation without successful outcomes and with negative outcomes
2	Options to sort and extract based on any coded characteristic, for instance, for different outcomes, in what context, for which type of behavioral intervention, type of intervention design, or categories of specific implementation determinants (e.g., high, medium, or low readiness)
3	Options to extract the most commonly successful combinations of different types of elements and characteristics. For instance, in context X, for intervention type Y, and population Z, what is the most commonly successful combination of consultation elements and other implementation strategies, and how are they most commonly carried out when they work (by whom, dosage, sequence or synchronicity, duration etc.)?
4	Statistical analyses such as meta-regressions can be used to estimate implementation or intervention effects with and without specific consultation practice elements and test associations between specific consultation practice elements, process elements and context variables
5	Component network meta-analysis can provide estimates of the relative effectiveness of consultation elements and different combinations of elements

## Advancing and Disseminating Common Elements in Implementation Science

Completing the algorithms described in Table 3 manually is rather cumbersome. However, the underlying logic can be computerized to make the sorting based on different configurations and patterns automated (Engell, 2021). Herein lies the potential for rapidly synthesizing the literature into pragmatic implications and predictions for research and practice. Artificial intelligence techniques such as Natural Language Processing (NLP) can further enhance the automation and usability of such systems (Olivetti et al., 2020). However, they rely on semi-standardized reporting of intervention and implementation details. Machine learning and other advanced analyses can be used to learn even more from such systems of big datasets, including using individual participant data to improve precision and idiosyncratic implications (e.g., Furukawa et al., 2021). In addition, feeding end-user data back into big data ecosystems can further improve predictions (Celi et al., 2020) where elements can be used as inputs into neural network architectures (Weng, 2020). For interested readers, the Human Behavior Change Project (Michie et al., 2020) is a pioneering example from behavior change science pursuing several of these possibilities.

There are existing dissemination tools available where common elements results can be useful. For example, evidence gap maps (EGMs) consolidate what we know and do not know from evaluation research about policies and practices, and visually present the results in interactive maps (example of Mega EGM by the Campbell Collaboration and UNICEF here; Saran et al., 2020). Similar maps can be made for evidence about common intervention and implementation elements, and, in time, may be integrated as subcategories of policies and practices in joint EGMs.

There are several online repositories and clearinghouses disseminating guides for evidence-based programs (EBPs), and such guides could also disseminate which implementation elements commonly work to implement and sustain the EBPs. PracticeWise (PracticeWise LLC, n.d.) is a commercial example of an online repository and clinical decision-making tool where users can find short guides for using common elements of EBPs for children’s mental health sorted by specific populations and contexts. Similar searchable repositories, preferably publicly available (e.g., through government funding), could in time be made for common elements of implementation and be integrated with evidence about EBPs. Although current formats for scientific publishing is predominantly manuscript-based, scientific publishing can (and likely will) evolve to other interactive and more real-time formats, such as, for instance, libraries and repositories with open datasets.

### Practical Application of Common Elements in Implementation Practice

Dissemination of common elements results offers advantages for pragmatic use. A primary practical application of common elements for implementation science could be co-designing implementation strategies in partnership with implementation researchers and stakeholders. Indeed, while recommendations have been made about what to report regarding implementation strategies (e.g., action, actor, goal, duration; Proctor et al., 2013), implementation strategies require tailoring and adaptations to fit within diverse contexts (Miller et al., 2021) and for specific intervention characteristics (Lyon et al., 2020a). As such, a common elements approach could allow for increased understanding of what “building blocks” are needed and influential when co-designing an implementation effort, with guidance as to which elements tend to be combined, what can be adapted, and what is likely required for successful outcomes in a given setting and process. Ideally, the elements used and the planned and ad hoc adaptations should be tracked to continue to enhance our understanding of the necessary ingredients to promote high-quality implementation (Finley et al., 2018; Miller et al., 2021).

Common elements can also provide implications for training and education in implementation science. A well-articulated benefit of common elements approaches is that, compared to knowledge in the form of complex models and programs, elements can be more easily learned, retained, and integrated with practitioners’ more fluid knowledge and expertise (Chen et al., 2021; Engell et al., 2021). Focusing the training of implementation practitioners and stakeholders on the implementation elements that commonly work (and discarding those that commonly do not work), both generically and context-specifically, may be an efficient approach to educate and train for breadth and depth in implementation expertise. Element-based training curricula also lends itself to stepwise, sequential, and needs-based approaches to training and implementation (Engell et al., 2021).

Common elements approaches can also be leveraged in system and service design (Chorpita et al., 2021). As mentioned in the introduction, the unit of an element (i.e., a “meaningful whole”) does not necessarily have to be an implementation strategy or an intervention. Elements can also be systems, organizations, and services, which can be downstream deconstructed into their parts (elements and components). Conversely, the construction or alterations of systems and implementation models can be informed by the parts that are commonly associated with desired determinants and outcomes. Chorpita and Daleiden’s (2014, 2018) work on models of coordinated strategic mental health systems are early examples along those lines of thinking.

## Limitations

Common elements reviews are limited by the studies available in the literature and the amount of details and data about implementation, intervention, and outcomes reported in those studies. Based on experience systematically reviewing thousands of studies, we are encouraged by movement in the field for researchers and authors to be substantially more attentive to reporting standards and details about intervention content and implementation processes. Another important limitation is the challenge of inferring element-level causality when the interventions or implementations tested “packages” of several elements (e.g., multi-faceted implementation strategy). Systematic common elements reviews are also subjected to the same biases as most traditional systematic reviews, particularly publication bias and the uncertain discrepancy between the intended experimental condition in primary studies (i.e., implementation manual) and the actual experimental condition (i.e., adherence to manual and quality of use). We also caution against *popularity bias*, which is the tendency of some elements to be frequently included in interventions and implementations based on popular opinion reasoning them as important, regardless of (unknown) effectiveness (Engell et al., 2020). Popularity bias may lead to certain elements commonly being “free agents” in effective interventions without necessarily contributing to their effects. Several methodological steps have been taken recent years to reduce these biases and improve inferences from element-level reviews of multi-element interventions (e.g., Engell et al., 2020; Furukawa et al., 2021; Solheim-Kvamme et al., 2022), and the precision will continue to improve with more use of reporting standards, developments in fidelity and process measurements, data availability, and advanced statistical analyses (Engell, 2021). For instance, computational linguistics and NLP will likely help improve the precision of fidelity measures (e.g., Flemotomos et al., 2021; Gallo et al., 2015; Imel et al., 2019), albeit there are challenges to overcome before such systems are in widespread use (Ahmadi et al., 2021). Nevertheless, highly common elements of effective interventions and implementations may best be described as *evidence-informed*, as they are derived from empirically tested interventions across contexts (i.e., informed by them), but not necessarily tested in isolation. They can also be viewed as hypothesis-generating for element-level experimental testing to establish evidence-based elements and mechanisms. At present, and as with all types of synthesized evidence, we recommend caution in interpreting common elements results and carefully evaluating their merit when they are considered for practice introduction.

## Recommendations for Next Steps

Our first recommendation is to *track and report element-level details in intervention and implementation studies.* It is essential to report all practices, processes, and context characteristics that are of relevance to the interventions and implementations, and report results from all proximal and distal outcomes measured. In other words, we need to capture data about how important elements and outcomes in interventions and implementations occur, unfold, and emerge in time and space. In addition to traditional data collection methods, this will likely include active and passive capturing of process data such as for instance ecological momentary assessments (EMA) and audio, geo, and bio feedback from devices and wearables (see Bettis et al., 2022 for review). Such detailed process data (e.g., flexible fidelity to functions; Engell, 2021; or fluctuating psychological processes; Russell & Gajos, 2020) are of particular importance for making more precise inferences about elements, mechanisms, and dynamic processes, and may benefit from adjustments or additions to current reporting standards. The use of EMA and other experience sampling methods have rapidly increased in psychosocial research the last decade and offers high ecological validity and low recall bias in information (Bettis et al., 2022; van Roekel et al., 2019). For instance, having EMA strategically coordinated with recordings of intervention sessions and information from wearables can provide detailed data on fidelity to intervention elements and proximal behavioral, emotional, and physical processes to index the dynamics of immediate and short-term responses to intervention. There are also practical, scientific, and ethical limitations related to real-time process data such as response burden, data noise, and health data privacy that requires mindful navigation.

We can use supplementary files to provide sufficient reporting of details and data, which can be used by NLP systems (or human coders). Follow current reporting standards and taxonomies, such as the ones referenced throughout the paper, when appropriate (for review of these and other relevant standards, see Rudd et al., 2020), provide explanations when deviating from current reporting standards and taxonomies, and add to them when relevant and appropriate. What we can learn from common elements reviews will be influenced by the theories and taxonomies researchers use in primary studies and in conducting reviews. We are unlikely to agree on one common ontology and taxonomy for each intervention and implementation phenomenon, nor should that necessarily be a goal. We should use current reporting standards and taxonomies to enable comparisons and integrations across studies and contexts. However, taxonomies operating as disciplinary standards must be subject to continuous debate, testing, and refinement, and intervention and implementation science should remain open to diverse and novel theory. While some differing ontological perspectives may be reconcilable in common theories and taxonomies, some may also require multiple taxonomies because of fundamentally different perspectives.

Our second recommendation is to *publish as many details about unsuccessful implementation as successful implementation*. As described in the step-by-step guide to common elements reviews in Table 3 and supplementary file 1, much can be learned from accumulating details about implementation failures and including data from unsuccessful implementation in our reviews, models, and analyses. Open journals like arXiv can facilitate reporting of unsuccessful implementation efforts that are vital to share but difficult to get published in peer-reviewed journals. The file drawer problem is an unethical waste of opportunity for scientific learning.

Our third recommendation is to *make data available when ethically appropriate.* Plan for data availability early to accommodate in applications to ethics boards. Use online repositories or create routines for sharing data with review researchers who inquire about data. Major publishers (e.g., Springer, Wiley, Elsevier) and funding institutions (e.g., National institute of Health in the US, National Institute for Health and Care Research in the UK) encourage and support data availability. The Research Council of Norway now require grant applications to include plans for data availability or provide a strong justification for why data cannot be publicly shared.

Our fourth recommendation is to *test common element-based hypotheses experimentally.* By testing hypotheses generated through advanced common elements reviews with different experimental designs (e.g., factorial trials, dismantling trials, time-series, natural experiments) we will both be testing the common elements concept as a theory and gain causal knowledge about the effects of elements and mechanisms in implementation. Complementing these trials with other methods (e.g., realist evaluation, system dynamics modeling, phenomenological studies) can help study processes, mechanisms, systems, and narratives from multiple perspectives to enrich our understanding of causal relations.

We call on implementation journals such as *Global Implementation Research and Applications, Implementation Science, Implementation Science Communications, Implementation Research and Practice* and other journals publishing implementation and intervention studies to accommodate these recommendations for authors.

## Conclusions

We proposed a research agenda for using advanced common elements methodology to improve the precision and pragmatism of implications from intervention and implementation research. For implementation science specifically, common elements methodologies are tools that can help synthesize and distill the literature into practical implementation applications, generate evidence-informed hypotheses about key elements, processes, and mechanisms in implementation, and promote evidence-informed precision tailoring of implementation strategies to contexts. We offered guidance and recommendations for researchers and journals on how we can realize those potentials, and we emphasized accommodating diverse approaches to understanding and studying implementation processes.

## Supplementary Information

Below is the link to the electronic supplementary material.

Supplementary file1 (DOCX 30 KB)

## Data Availability

Data sharing not applicable – no new data generated.

## References

[CR1] Abry T, Hulleman CS, Rimm-Kaufman SE (2015). Using indices of fidelity to intervention core components to identify program active ingredients. American Journal of Evaluation.

[CR2] Ahmadi A, Noetel M, Schellekens M, Parker P, Antczak D, Beauchamp M, Dicke T, Diezmann C, Maeder A, Ntoumanis N (2021). A systematic review of machine learning for assessment and feedback of treatment fidelity. Psychosocial Intervention.

[CR3] Albers B, Metz A, Burke K, Bührmann L, Bartley L, Driessen P, Varsi C (2021). Implementation support skills: Findings from a systematic integrative review. Research on Social Work Practice.

[CR4] Anjum RL, Mumford S (2018). What tends to be: The philosophy of dispositional modality.

[CR5] Assink M, Wibbelink CJ (2016). Fitting three-level meta-analytic models in R: A step-by-step tutorial. The Quantitative Methods for Psychology.

[CR6] Bækken, M. (2021). *What seems to be working here? Identifying common elements in brief emotion regulation interventions for children and adolescents–a systematic review* (Master’s thesis). University of Oslo, Norway. Retrieved from http://urn.nb.no/URN:NBN:no-90376

[CR7] Barth RP, Kolivoski KM, Lindsey MA, Lee BR, Collins KS (2014). Translating the common elements approach: Social work’s experiences in education, practice, and research. Journal of Clinical Child & Adolescent Psychology.

[CR8] Barth RP, Rozeff LJ, Kerns SEU, Baldwin MJ (2020). Partnering for success: Implementing a cross-systems collaborative model between behavioral health and child welfare. Children and Youth Services Review.

[CR9] Baumgartner M, Ambühl M (2020). Causal modeling with multi-value and fuzzy-set coincidence analysis. Political Science Research and Methods.

[CR10] Bettis AH, Burke TA, Nesi J, Liu RT (2022). Digital technologies for emotion-regulation assessment and intervention: A conceptual review. Clinical Psychological Science.

[CR11] Bolton P, Lee C, Haroz EE, Murray L, Dorsey S, Robinson C, Ugueto AM, Bass J (2014). A transdiagnostic community-based mental health treatment for comorbid disorders: Development and outcomes of a randomized controlled trial among Burmese refugees in Thailand. PLoS Medicine.

[CR12] Braithwaite J, Churruca K, Long JC, Ellis LA, Herkes J (2018). When complexity science meets implementation science: A theoretical and empirical analysis of systems change. BMC Medicine.

[CR13] Brown B, Gude WT, Blakeman T, van der Veer SN, Ivers N, Francis JJ, Lorencatto F, Presseau J, Peek N, Daker-White G (2019). Clinical performance feedback intervention theory (CP-FIT): A new theory for designing, implementing, and evaluating feedback in health care based on a systematic review and meta-synthesis of qualitative research. Implementation Science.

[CR14] Cane J, O’Connor D, Michie S (2012). Validation of the theoretical domains framework for use in behaviour change and implementation research. Implementation Science.

[CR15] Cashin AG, McAuley JH, Lee H (2022). Advancing the reporting of mechanisms in implementation science: A guideline for reporting mediation analyses (AGReMA). Implementation Research and Practice.

[CR16] Celi LA, Majumder MS, Ordóñez P, Osorio JS, Paik KE, Somai M (2020). Leveraging data science for global health.

[CR17] Centre for Evidence and Implementation. (2020). *A common elements approach to service provision for children and families in South Australia*. Centre for Evidence and Implementation. https://dhs.sa.gov.au/__data/assets/pdf_file/0014/90032/Common-elements-approach-service-provision-SA.pdf

[CR18] Chen C-C, Sutherland KS, Kunemund R, Sterrett B, Wilkinson S, Brown C, Maggin DM (2021). Intensifying interventions for students with emotional and behavioral difficulties: A conceptual synthesis of practice elements and adaptive expertise. Journal of Emotional and Behavioral Disorders.

[CR19] Chorpita BF, Becker KD, Daleiden EL (2007). Understanding the common elements of evidence-based practice. Journal of the American Academy of Child & Adolescent Psychiatry.

[CR20] Chorpita BF, Daleiden EL (2009). Mapping evidence-based treatments for children and adolescents: Application of the distillation and matching model to 615 treatments from 322 randomized trials. Journal of Consulting and Clinical Psychology.

[CR21] Chorpita BF, Daleiden EL (2014). Structuring the collaboration of science and service in pursuit of a shared vision. Journal of Clinical Child & Adolescent Psychology.

[CR22] Chorpita BF, Daleiden EL (2018). Coordinated strategic action: Aspiring to wisdom in mental health service systems. Clinical Psychology: Science and Practice.

[CR23] Chorpita BF, Daleiden EL, Vera JD, Guan K (2021). Creating a prepared mental health workforce: Comparative illustrations of implementation strategies. Evidence- Based Mental Health.

[CR24] Chorpita BF, Daleiden EL, Weisz JR (2005). Identifying and selecting the common elements of evidence based interventions: A distillation and matching model. Mental Health Services Research.

[CR25] Chorpita BF, Rotheram-Borus MJ, Daleiden EL, Bernstein A, Cromley T, Swendeman D, Regan J (2011). The old solutions are the new problem: How do we better use what we already know about reducing the burden of mental illness?. Perspectives on Psychological Science.

[CR26] Conroy MA, Sutherland KS, Algina J, Ladwig C, Werch B, Martinez J, Jessee G, Gyure M (2019). Outcomes of the BEST in CLASS intervention on teachers’ use of effective practices, self-efficacy, and classroom quality. School Psychology Review.

[CR27] Curran GM (2020). Implementation science made too simple: A teaching tool. Implementation Science Communications.

[CR28] Damschroder LJ, Aron DC, Keith RE, Kirsh SR, Alexander JA, Lowery JC (2009). Fostering implementation of health services research findings into practice: A consolidated framework for advancing implementation science. Implementation Science.

[CR29] Embry DD, Biglan A (2008). Evidence-based kernels: Fundamental units of behavioral influence. Clinical Child and Family Psychology Review.

[CR30] Engell, T. (2021). Co-design and implementation of common elements-based academic support in Norwegian Child Welfare Services. *Dissertation*. Retrieved from http://urn.nb.no/URN:NBN:no-89115

[CR31] Engell T, Kirkøen B, Hammerstrøm KT, Kornør H, Ludvigsen KH, Hagen KA (2020). Common elements of practice, process and implementation in out-of-school-time academic interventions for at-risk children: A systematic review. Prevention Science.

[CR32] Engell T, Løvstad AM, Kirkøen B, Ogden T, Amlund Hagen K (2021). Exploring how intervention characteristics affect implementability: A mixed methods case study of common elements-based academic support in child welfare services. Children and Youth Services Review.

[CR33] Eyberg SM, Funderburk B (2011). Parent-child interaction therapy protocol.

[CR34] Finley EP, Huynh AK, Farmer MM, Bean-Mayberry B, Moin T, Oishi SM, Moreau JL, Dyer KE, Lanham HJ, Leykum L (2018). Periodic reflections: A method of guided discussions for documenting implementation phenomena. BMC Medical Research Methodology.

[CR35] Fixsen, D. L., Blase, K., Friedman, R. M., & Wallace, F. (2005). *Implementation research: A synthesis of literature*. University of South Florida, Louis de la Parte Florida Mental Health Institute, National Implementation Research Network. Retrieved from https://nirn.fpg.unc.edu/resources/implementation-research-synthesis-literature

[CR36] Flemotomos, N., Martinez, V. R., Chen, Z., Singla, K., Ardulov, V., Peri, R., Caperton, D. D., Gibson, J., Tanana, M. J., & Georgiou, P. G. (2021). Am I a good therapist? Automated evaluation of psychotherapy skills using speech and language technologies. *CoRR, Abs/2102.11265*. 10.3758/s13428-021-01623-4 10.3758/s13428-021-01623-4 PMC8810915 34346043

[CR37] Furukawa TA, Suganuma A, Ostinelli EG, Andersson G, Beevers CG, Shumake J, Berger T, Boele FW, Buntrock C, Carlbring P, Choi I, Christensen H, Mackinnon A, Dahne J, Huibers MJH, Ebert DD, Farrer L, Forand NR, Strunk DR (2021). Dismantling, optimising, and personalising internet cognitive behavioural therapy for depression: A systematic review and component network meta-analysis using individual participant data. The Lancet Psychiatry.

[CR38] Gallo C, Pantin H, Villamar J, Prado G, Tapia M, Ogihara M, Cruden G, Brown CH (2015). Blending qualitative and computational linguistics methods for fidelity assessment: Experience with the familias unidas preventive intervention. Administration and Policy in Mental Health and Mental Health Services Research.

[CR39] Garland AF, Hawley KM, Brookman-Frazee L, Hurlburt MS (2008). Identifying common elements of evidence-based psychosocial treatments for children’s disruptive behavior problems. Journal of the American Academy of Child & Adolescent Psychiatry.

[CR40] Greenhalgh T, Robert G, Macfarlane F, Bate P, Kyriakidou O (2004). Diffusion of innovations in service organizations: Systematic review and recommendations. The Milbank Quarterly.

[CR41] Hawe P, Shiell A, Riley T (2004). Complex interventions: How “out of control” can a randomised controlled trial be?. BMJ.

[CR42] Heinonen E, Nissen-Lie HA (2019). The professional and personal characteristics of effective psychotherapists: A systematic review. Psychotherapy Research.

[CR43] Helland SS, Kirkøen B, Espenes K, Melblom A, Engell T, Wentzel-Larsen T, Kjøbli J (2022). Elements in mental health interventions associated with effects on emotion regulation in adolescents: A meta-analysis. Administration and Policy in Mental Health and Mental Health Services Research.

[CR44] Higgins JP, Green S (2011). Cochrane handbook for systematic reviews of interventions.

[CR45] Hill CE, Spiegel SB, Hoffman MA, Kivlighan DM, Gelso CJ (2017). Therapist expertise in psychotherapy revisited ψ. The Counseling Psychologist.

[CR46] Hoffmann TC, Glasziou PP, Boutron I, Milne R, Perera R, Moher D, Altman DG, Barbour V, Macdonald H, Johnston M, Lamb SE, Dixon-Woods M, McCulloch P, Wyatt JC, Chan A-W, Michie S (2014). Better reporting of interventions: Template for intervention description and replication (TIDieR) checklist and guide. BMJ.

[CR47] Hofmann SG, Hayes SC (2019). The future of intervention science: Process-based therapy. Clinical Psychological Science.

[CR48] Hogue A, Bobek M, Dauber S, Henderson CE, McLeod BD, Southam-Gerow MA (2019). Core elements of family therapy for adolescent behavior problems: Empirical distillation of three manualized treatments. Journal of Clinical Child & Adolescent Psychology.

[CR49] Huibers MJH, Lorenzo-Luaces L, Cuijpers P, Kazantzis N (2021). On the road to personalized psychotherapy: A research agenda based on cognitive behavior therapy for depression. Frontiers in Psychiatry.

[CR50] Imel ZE, Pace BT, Soma CS, Tanana M, Hirsch T, Gibson J, Georgiou P, Narayanan S, Atkins DC (2019). Design feasibility of an automated, machine-learning based feedback system for motivational interviewing. Psychotherapy.

[CR51] Ivers N, Jamtvedt G, Flottorp S, Young JM, Odgaard-Jensen J, French SD, O’Brien MA, Johansen M, Grimshaw J, Oxman AD (2012). Audit and feedback: Effects on professional practice and healthcare outcomes. Cochrane Database of Systematic Reviews.

[CR52] Kazdin AE (2009). Understanding how and why psychotherapy leads to change. Psychotherapy Research.

[CR53] Leijten P, Gardner F, Melendez-Torres GJ, van Aar J, Hutchings J, Schulz S, Knerr W, Overbeek G (2019). Meta-analyses: Key parenting program components for disruptive child behavior. Journal of the American Academy of Child & Adolescent Psychiatry.

[CR54] Leijten P, Weisz JR, Gardner F (2021). Research strategies to discern active psychological therapy components: A scoping review. Clinical Psychological Science.

[CR55] Lewis CC, Klasnja P, Powell BJ, Lyon AR, Tuzzio L, Jones S, Walsh-Bailey C, Weiner B (2018). From classification to causality: advancing understanding of mechanisms of change in implementation science. Frontiers in Public Health.

[CR56] Lyon AR, Brewer SK, Areán PA (2020). Leveraging human-centered design to implement modern psychological science: Return on an early investment. American Psychologist.

[CR57] Lyon AR, Comtois KA, Kerns SE, Landes SJ, Lewis CC (2020). Closing the science–practice gap in implementation before it widens. Implementation Science.

[CR58] McLeod BD, Sutherland KS, Martinez RG, Conroy MA, Snyder PA, Southam-Gerow MA (2017). Identifying common practice elements to improve social, emotional, and behavioral outcomes of young children in early childhood classrooms. Prevention Science.

[CR59] Mellblom, A. V., Helland, S.S., Engell, T., Bergseth, J., Solheim Kvamme, L., Mørk, A. S., Bækken, M., Kjøbli, J. (2023). Common elements in brief interventions on emotion regulation in adolescents: A systematic review and meta-analysis. *Submitted for publication 03.01.2023*

[CR60] Metz A, Albers B, Burke K, Bartley L, Louison L, Ward C, Farley A (2021). Implementation practice in human service systems: Understanding the principles and competencies of professionals who support implementation. Human Service Organizations: Management, Leadership & Governance.

[CR61] Michie S, Richardson M, Johnston M, Abraham C, Francis J, Hardeman W, Eccles MP, Cane J, Wood CE (2013). The behavior change technique taxonomy (v1) of 93 hierarchically clustered techniques: Building an international consensus for the reporting of behavior change interventions. Annals of Behavioral Medicine.

[CR62] Michie S, Thomas J, Aonghusa PM, West R, Johnston M, Kelly MP, Shawe-Taylor J, Hastings J, Bonin F, O’Mara-Eves A (2020). The human behaviour-change project: An artificial intelligence system to answer questions about changing behaviour. Wellcome Open Research.

[CR63] Miller CJ, Barnett ML, Baumann AA, Gutner CA, Wiltsey-Stirman S (2021). The FRAME-IS: A framework for documenting modifications to implementation strategies in healthcare. Implementation Science.

[CR97] Moullin JC, Dickson KS, Stadnick NA (2019). Systematic review of the Exploration, Preparation, Implementation, Sustainment (EPIS) framework. Implementation Science.

[CR64] Mulder R, Murray G, Rucklidge J (2017). Common versus specific factors in psychotherapy: Opening the black box. The Lancet Psychiatry.

[CR65] Murray LK, Haroz E, Dorsey S, Kane J, Bolton PA, Pullmann MD (2020). Understanding mechanisms of change: An unpacking study of the evidence-based common-elements treatment approach (CETA) in low and middle income countries. Behaviour Research and Therapy.

[CR66] Nadeem E, Gleacher A, Beidas RS (2013). Consultation as an implementation strategy for evidence-based practices across multiple contexts: Unpacking the black box. Administration and Policy in Mental Health and Mental Health Services Research.

[CR67] Nilsen P, Bernhardsson S (2019). Context matters in implementation science: A scoping review of determinant frameworks that describe contextual determinants for implementation outcomes. BMC Health Services Research.

[CR68] Okamura KH, Orimoto TE, Nakamura BJ, Chang B, Chorpita BF, Beidas RS (2020). A history of child and adolescent treatment through a distillation lens: Looking back to move forward. The Journal of Behavioral Health Services & Research.

[CR69] Olivetti EA, Cole JM, Kim E, Kononova O, Ceder G, Han TYJ, Hiszpanski AM (2020). Data-driven materials research enabled by natural language processing and information extraction. Applied Physics Reviews.

[CR70] Park AL, Rith-Najarian LR, Saifan D, Gellatly R, Huey SJ, Chorpita BF (2022). Strategies for incorporating culture into psychosocial interventions for youth of color. Evidence-Based Practice in Child and Adolescent Mental Health.

[CR71] Pinnock H, Barwick M, Carpenter CR, Eldridge S, Grandes G, Griffiths CJ, Rycroft-Malone J, Meissner P, Murray E, Patel A, Sheikh A, Taylor SJC (2017). Standards for reporting implementation studies (StaRI) statement. BMJ.

[CR72] Pompoli A, Furukawa TA, Efthimiou O, Imai H, Tajika A, Salanti G (2018). Dismantling cognitive-behaviour therapy for panic disorder: A systematic review and component network meta-analysis. Psychological Medicine.

[CR73] Powell BJ, Waltz TJ, Chinman MJ, Damschroder LJ, Smith JL, Matthieu MM, Proctor EK, Kirchner JE (2015). A refined compilation of implementation strategies: Results from the Expert Recommendations for Implementing Change (ERIC) project. Implementation Science.

[CR74] PracticeWise LLC. (n.d.). *PracticeWise evidence-based searchable database*. PracticeWise LLC. Retrieved August 20, 2021, from https://www.practicewise.com/

[CR75] Presseau J, McCleary N, Lorencatto F, Patey AM, Grimshaw JM, Francis JJ (2019). Action, actor, context, target, time (AACTT): A framework for specifying behaviour. Implementation Science.

[CR76] Proctor EK, Powell BJ, McMillen JC (2013). Implementation strategies: Recommendations for specifying and reporting. Implementation Science.

[CR77] Rudd BN, Davis M, Beidas RS (2020). Integrating implementation science in clinical research to maximize public health impact: A call for the reporting and alignment of implementation strategy use with implementation outcomes in clinical research. Implementation Science.

[CR78] Russell MA, Gajos JM (2020). Annual research review: Ecological momentary assessment studies in child psychology and psychiatry. Journal of Child Psychology and Psychiatry.

[CR79] Saran A, White H, Albright K, Adona J (2020). Mega-map of systematic reviews and evidence and gap maps on the interventions to improve child well-being in low-and middle-income countries. Campbell Systematic Reviews.

[CR80] Seide SE, Jensen K, Kieser M (2020). A comparison of Bayesian and frequentist methods in random-effects network meta-analysis of binary data. Research Synthesis Methods.

[CR81] Solheim-Kvamme L, Nes SK, Nes RB, Vaskinn L, Waaler PM, Wentzel-Larsen T, Kjøbli J (2022). Common practice elements in treatment programs for adolescents with externalizing and internalizing problems: A meta-analysis. Residential Treatment for Children & Youth.

[CR82] Sterman, J. (2002). System Dynamics: systems thinking and modeling for a complex world. ESD Working Papers; ESD-WP-2003–01.13-ESD Internal Symposium.

[CR83] Tugendrajch SK, Sheerin KM, Andrews JH, Reimers R, Marriott BR, Cho E, Hawley KM (2021). What is the evidence for supervision best practices?. The Clinical Supervisor.

[CR84] Tuti T, Nzinga J, Njoroge M, Brown B, Peek N, English M, Paton C, van der Veer SN (2017). A systematic review of electronic audit and feedback: Intervention effectiveness and use of behaviour change theory. Implementation Science.

[CR85] van der Pol TM, van Domburgh L, van Widenfelt BM, Hurlburt MS, Garland AF, Vermeiren RR (2019). Common elements of evidence-based systemic treatments for adolescents with disruptive behaviour problems. The Lancet Psychiatry.

[CR86] van Roekel E, Keijsers L, Chung JM (2019). A review of current ambulatory assessment studies in adolescent samples and practical recommendations. Journal of Research on Adolescence.

[CR87] Varzi AC (1996). Parts, wholes, and part-whole relations: The prospects of mereotopology. Data & Knowledge Engineering.

[CR88] Wagner A (1999). Causality in complex systems. Biology and Philosophy.

[CR89] Waltz TJ, Powell BJ, Matthieu MM, Damschroder LJ, Chinman MJ, Smith JL, Proctor EK, Kirchner JE (2015). Use of concept mapping to characterize relationships among implementation strategies and assess their feasibility and importance: Results from the expert recommendations for implementing change (ERIC) study. Implementation Science.

[CR90] Wampold BE (2015). How important are the common factors in psychotherapy? An update. World Psychiatry.

[CR91] Weiss WM, Murray LK, Zangana GAS, Mahmooth Z, Kaysen D, Dorsey S, Lindgren K, Gross A, Murray SM, Bass JK, Bolton P (2015). Community-based mental health treatments for survivors of torture and militant attacks in Southern Iraq: A randomized control trial. BMC Psychiatry.

[CR92] Weisz JR, Chorpita BF, Palinkas LA, Schoenwald SK, Miranda J, Bearman SK, Daleiden EL, Ugueto AM, Ho A, Martin J (2012). Testing standard and modular designs for psychotherapy treating depression, anxiety, and conduct problems in youth: A randomized effectiveness trial. Archives of General Psychiatry.

[CR93] Weng, W. H. (2020). Machine learning for clinical predictive analytics. In *Leveraging data science for global health* (pp. 199–217). Springer.

[CR94] Wentzel-Larsen, T. (2021). *Code for a shiny app for three-level meta-analysis*. Retrieved from https://github.com/ToreWentzel-Larsen/threelevel

[CR95] Westerlund A, Nilsen P, Sundberg L (2019). Implementation of implementation science knowledge: The research-practice gap paradox. Worldviews on Evidence-Based Nursing.

[CR98] Wiltsey Stirman, S., & Comer, J. S. (2018). What are we even trying to implement? Considering the relative merits of promoting evidence-based protocols, principles, practices, or policies. *Clinical Psychology: Science and Practice,**25*(4).

[CR96] Winje, H. N. (2019). *Identifisering av felleselementer fra relasjonelle intervensjoner for omsorgssvikt og mishandling* (Master’s thesis). Univeristy of Oslo. Retrieved from http://urn.nb.no/URN:NBN:no-73472

