# Supplementary material for: Common Elements Approaches to Implementation Research and Practice: Methods and Integration with Intervention Science
Source: Glob Implement Res Appl. 2023 Mar 22;3(1):1–15. doi: 10.1007/s43477-023-00077-4 (PMC10063479; doi:10.1007/s43477-023-00077-4)
Supplement: Supplementary file 1 — Supplementary file1 (DOCX 30 KB) [file 43477_2023_77_MOESM1_ESM.docx]

**Supplementary file 1**

**Common Elements Approaches to Implementation Research and Practice: Methods and Integration with Intervention Science**

Engell, Stadnick, Aarons & Barnett (2023)

*Global Implementation Research and Applications*

Corresponding author:

Thomas Engell, [te@r-bup.no](mailto:te@r-bup.no)

Regional Centre for Child and Adolescent Mental Health, Eastern and Southern Norway

**Step-by-step description of systematic common elements review methodology**

| Step | Description |
| --- | --- |
| 1 | **Study selection from systematic reviews or databases**   - We define criteria for identifying studies to code in common elements matrixes or databases. Criteria typically defines the populations, interventions and implementation strategies, comparisons, and outcomes of interest. - We identify studies through conducting systematic reviews or using existing reviews, reviews, databases or repositories of studies assessed for quality and risk of bias. The critical point is that the literature search and selection process and quality assessments meet the standards of high-quality systematic reviews to limit biases. We direct readers to the Cochrane Handbook for Systematic Reviews of Interventions (Higgins & Green, 2011) for guidance. When using a selection of studies based on prior reviews, we can use the AMSTAR checklist to assess the quality of reviews (Shea et al., 2017). - Using external consultation as an example implementation strategy of interest, we could, for instance, identify or conduct systematic reviews of experimental studies where external consultation was either experimentally tested as an implementation strategy or used as an implementation strategy in an experimental study of the implementation of one or more psychosocial interventions, for any setting and profession providing psychosocial help to any population. |
| 2 | **Gather material, information, and data from included studies**   - We gather as much relevant information about the studies and their experimental conditions as possible. This can include but is not limited to scientific papers and supplementary files, intervention manuals, implementation plans, data openly shared from the study, dissertations, research reports, and grey literature. - This step often includes contacting authors with requests for details and data about interventions, implementations, and outcomes. |
| 3 | **Prepare and pilot the coding system**   - After reviewing a sample of studies, we specify and define all elements and components we anticipate are relevant for the review and other outcomes and determinants of interest. Unambiguous definitions are important to promote ease in coding and reliability. The deconstruction explained on page 8 in the article is central to this process and includes making conscious decisions about theory. - Using existing theories in the form of ontologies and taxonomies can be useful and save time. However, additional conceptual work is often needed – especially if sufficient information is available in studies to warrant a highly deconstructed level of detail in coding, and keep in mind that choosing one taxonomy is a theoretical choice that shapes the results. - We recommend doing this conceptual work as an iterative data-driven process of prototyping (with stakeholders if appropriate), piloting for construct validity and coding reliability, revising, and repeating and being inclusive rather than exclusive about elements/codes at this stage. - We also need to prepare coding matrices, a database, or other systems that will organize and store the coded information appropriately for conducting planned algorithms and analyses. A key feature needed for the method we present here is a network architecture (i.e., the ability to track each code and each combination or branch of codes back to its origin study and its specific outcomes) while at the same time collate codes from several studies together. See the coding manual in Engell et al. (2020) for step-by-step guidance using matrices in Microsoft Excel. In addition, having the functionality to provide values, weights, or conditions to codes can be useful, for instance, for scaling outcomes such as fidelity or process elements such as dosage. Similar coding can be done in statistical software (e.g., Helland et al., 2022), and it is possible to design more user-friendly coding platforms using data management software such as Confirmit or Qualtrics. - We also prepare for the opportunity to add unanticipated codes during coding when the coding team agrees it should be added, and we train coders to scan material for inductive codes in addition to deductive codes. Inductive coding can help identify potentially meaningful novelties that do not fit existing theories and taxonomies and reduce biases towards “popular” elements (Engell et al., 2020). |
| 4 | **Coding iterations**   - All studies are coded by at least two independent coders, and coding conflicts are resolved through discussions or with a supervisor. Coding can be labor intensive, and we tend to form teams of several coding pairs. - Using the material available (e.g., papers, manuals, data), coders code each study for all outcomes, elements and components (practice, process, context), and other study characteristics and determinants of interest with unique traceable IDs in coding matrices. Then they code and calculate data of interest needed to run statistical analyses (e.g., effect sizes, fidelity data). Tips to ease usability in coding is available in the manual by Engell et al. (2020). - Coding metrics are recorded to test coding reliability. |
| 5 | **Apply algorithms to identify common elements and combinations**   - In this step, frequency-based algorithms appropriate for the data matrix or database are used to identify common elements and common combinations of elements. The algorithms can be applied across the whole dataset, such as choosing intervention fidelity as an outcome and identifying the implementation elements and combinations of elements that are most commonly used in high fidelity implementations adjusted for use in low fidelity implementations. - Algorithms can also be applied for specific configurations of elements such as, for instance, restricted to studies where external consultation was used as an implementation strategy for transdiagnostic child mental health interventions with fidelity and effectiveness as outcome measures. We tend to apply algorithms in the following order:  1. Calculate adjusted frequency values of common elements for each outcome of interest (e.g., inclusion in effective interventions/implementations accounted for inclusion in ineffective and iatrogenic) 2. Calculate adjusted frequency values of common combinations of elements, components, and characteristics for each outcome of interest 3. Calculate adjusted frequency values for specific configurations of interest (e.g., specific target group, context, and outcome) 4. Visualize results to ease interpretation (e.g., color code elements and combinations based on frequency values, extract to tables) 5. Formulate hypotheses based on common elements, combinations, and configurations  - To formulate hypotheses and/or implications about common elements or configurations we calculate the commonness (i.e., adjusted frequency values) of the most common elements and combinations we identify, adjust based on any weights used (e.g., risk of bias or adverse outcomes; Engell et al., 2020), and apply criteria for what qualifies as being common enough based on the literature or convenience (e.g., 25% most common elements or combinations; Engell et al., 2020). We can also statistically test which elements that are significantly more common than others (see Solheim-Kvamme et al., 2022, for example). |
| 6 | **Statistical analyses**   - A number of statistical analyses can be used to exploratory test the common elements data, test hypotheses informed by step 5, and triangulate results for interpretation. See Leijten et al. (2021) for review of typical analytical strategies. - We first extract the necessary data from the matrixes and organize them for the analyses of choice, and calculating effects sizes are often necessary (e.g., Helland et al, 2022). - We can use three-level meta-regression analyses to test how the inclusion and exclusion of common elements and common configurations are associated with effect sizes for different outcomes and how other elements moderate associations. To conduct these analyses, we use a freely available shiny app in R developed by Wentzel-Larsen (Wentzel-Larsen, 2021, available [here](https://github.com/ToreWentzel-Larsen/threelevel)) A complete step-by-step guide to such meta-analytic models have been provided by Assink and Wibbelink (2016), upon which the shiny app is based. Examples of similar approaches and analytic strategies can be found in Leijten et al.’s (2019) study of elements in parenting programs for disruptive child behavior or Eckerstorfer et al.’s (2018) study of common elements of mobile technology to increase physical activity. - Component Network Meta-Analysis (cNMA) can estimate the relative effectiveness of elements and combinations across studies. cNMA assumes the effects of composite interventions/implementations are the sum of their elements (see page 8 for relevant theories about meronymic relations) and can be conducted using Bayesian statistics or frequentist methods (Seide et al., 2020). See Pompoli et al. (2018) for analytic strategy and example application. - Another analysis that can be useful for common elements reviews is coincidence analyses (CNA, Baumgartner & Ambühl, 2020), a configurational-comparative method for testing causal regularities that was recently introduced to implementation science (Whitaker et al., 2020). We caution that we have not used this analysis ourselves yet. However, the logic and algorithms applied to coding combinations of elements as we present in this paper seems to make CNA fit for testing causal regularities based on set theory combinations (i.e., conjunction, disjunction, and negation) of coded elements and characteristics. - The perhaps most promising application of statistical analyses in this step is likely collating mega-reviews or continuously feed coded data into big data ecosystems with network architecture designed for machine learning. The application of machine learning and other artificial intelligence (AI) methods is discussed later in the article. |

**References**

Assink, M., & Wibbelink, C. J. (2016). Fitting three-level meta-analytic models in R: A step-by-step tutorial. *The Quantitative Methods for Psychology*, *12*(3), 154-174.

Baumgartner, M., & Ambühl, M. (2020). Causal Modeling with Multi-Value and Fuzzy-Set Coincidence Analysis. *Political Science Research and Methods* 8, 526-542, doi: 10.1017/psrm.2018.45

Eckerstorfer, L. V., Tanzer, N. K., Vogrincic-Haselbacher, C., Kedia, G., Brohmer, H., Dinslaken, I., & Corcoran, K. (2018). Key Elements of mHealth Interventions to Successfully Increase Physical Activity: Meta-Regression. *JMIR MHealth and UHealth*, *6*(11), e10076. https://doi.org/10.2196/10076

Engell, T., Kirkøen, B., Hammerstrøm, K. T., Kornør, H., Ludvigsen, K. H., & Hagen, K. A. (2020). Common elements of practice, process and implementation in out-of-school-time academic interventions for at-risk children: A systematic review. *Prevention Science*, *21*(4), 545-556.

Helland, S. S., Mellblom, A. V., Kjøbli, J., Wentzel-Larsen, T., Espenes, K., Engell, T., & Kirkøen, B. (2022). Elements in mental health interventions associated with effects on emotion regulation in adolescents: a meta-analysis. *Administration and Policy in Mental Health and Mental Health Services Research*, *49*(6), 1004-1018.

Higgins, J. P., & Green, S. (Eds.). (2011). Cochrane handbook for systematic reviews of interventions (vol. 4). John Wiley & Sons.

Kvamme, L. S., Keles, S., Nes, R. B., Vaskinn, L., Waaler, P. M., Wentzel-Larsen, T., & Kjøbli, J. (2022). Common Practice Elements in Treatment Programs for Adolescents with Externalizing and Internalizing Problems: A Meta-Analysis. *Residential Treatment for Children & Youth*, 1-31.

Leijten, P., Gardner, F., Melendez-Torres, G. J., van Aar, J., Hutchings, J., Schulz, S., Knerr, W., & Overbeek, G. (2019). Meta-Analyses: Key Parenting Program Components for Disruptive Child Behavior. *Journal of the American Academy of Child & Adolescent Psychiatry*, *58*(2), 180–190. https://doi.org/10.1016/j.jaac.2018.07.900

Leijten, P., Weisz, J. R., & Gardner, F. (2021). Research Strategies to Discern Active Psychological Therapy Components: A Scoping Review. *Clinical Psychological Science*, *9*(3), 307–322.

Pompoli, A., Furukawa, T. A., Efthimiou, O., Imai, H., Tajika, A., & Salanti, G. (2018). Dismantling cognitive-behaviour therapy for panic disorder: a systematic review and component network meta-analysis. *Psychological medicine*, *48*(12), 1945-1953.

Shea, B. J., Reeves, B. C., Wells, G., Thuku, M., Hamel, C., Moran, J., ... & Henry, D. A. (2017). AMSTAR 2: a critical appraisal tool for systematic reviews that include randomised or non-randomised studies of healthcare interventions, or both. *bmj*, *358*.

Wentzel-Larsen, T. (2021). *Code for a shiny app for three-level meta-analysis*. https://github.com/ToreWentzel-Larsen/threelevel

Whitaker, R. G., Sperber, N., Baumgartner, M., Thiem, A., Cragun, D., Damschroder, L., ... & Birken, S. (2020). Coincidence analysis: a new method for causal inference in implementation science. *Implementation Science*, *15*(1), 1-10.
